# Early Life Stress and Body-Mass Index Modulate Brain Connectivity in Alcohol Use Disorder

**DOI:** 10.21203/rs.3.rs-3150110/v1

**Published:** 2023-07-20

**Authors:** Khushbu Agarwal, Paule Joseph, Rui Zhang, Melanie Schwandt, Vijay Ramchandani, Nancy Diazgranados, David Goldman, Reza Momenan

**Affiliations:** National Institutes of Health; National Institutes of Health; National Institute on Alcohol Abuse and Alcoholism; National Institute on Alcohol Abuse and Alcoholism / National Institutes of Health; National Institutes of Health; NIH; National Institutes of Health; National Institute On Alcohol Abuse and Alcoholism

**Keywords:** Early life stress, Alcohol use disorder, Body mass index, Resting state functional MRI, Seed-based voxel connectivity, Salience network

## Abstract

Early life stress (ELS) significantly increases susceptibility to alcohol use disorder (AUD) by affecting the interplay between executive and salience networks (SN). The link between AUD and higher body-mass index (BMI) is known, but we lack understanding of how BMI impacts the relationship between ELS and brain connectivity in individuals with AUD. To bridge this gap, we investigated the effects of ELS on brain connectivity in AUD participants, taking into account differences in BMI. The cohort included 401 individuals with AUD, with approximately 60% having a BMI ≥ 25. Within the overall cohort, 123 participants underwent resting-state functional magnetic resonance imaging, revealing intriguing anticorrelations between SN seeds and brain regions involved in somatosensory processing, motor coordination, and executive control as an effect of ELS. Examining the relationship between ELS-driven brain connectivity and BMI, we observed negative correlations in connectivity among low BMI (≤ 24.9) vs. high BMI (≥ 25) individuals. For example, the left supramarginal gyrus (SMG) seed exhibited decreased connectivity with emotion regulation and decision-making regions, including the right occipital cortex, posterior cingulate cortex, and precuneus clusters (all |β| < −0.03, |p| < 0.05). Additionally, the right SMG seed showed reduced connectivity with impulse control and executive function regions, such as the left postcentral/middle frontal gyrus cluster (β = 0.04, p = 0.02). These findings highlight the role of ELS-induced alterations in SN seed connectivity, influenced by BMI, in the neurobiology of AUD. Understanding the neural mechanisms linking obesity, AUD, and ELS can guide targeted interventions for this population.

## Introduction

Early life stress (ELS), as determined by self-reported traumatic childhood events, such as emotional abuse, severe family conflict, domestic violence and bullying, has been linked to increased vulnerability to the development of alcohol use disorder (AUD) ^1,2,3^. Individuals with AUD and obesity exhibit poor decision-making abilities, which has been linked to disruptions in the salience network (SN) assessed in resting state functional MRI studies ^4, 5^. This maladaptive decision-making has been attributed to difficulties in switching between executive and salience networks ^6^. Accordingly, studies have extensively examined the relationship between heavy alcohol consumption and increased body weight over the past years ^7^.

It has been speculated that heavy alcohol consumption can lead to a higher body-mass index (BMI) via insulin resistance ^8, 9^. Although the evidence for a comorbidity between obesity and AUD is conflicting ^10^, the clinical relationship between these two disorders is complex and both are affected by common aspects of vulnerability to adverse events, as well as subsequent events whereby excess alcohol consumption can lead to both weight gain and to weight loss ^7, 11, 12^. Previous studies have shown that ELS is associated alterations in brain structure and function, including reduced *centrality* (defined as the impact of a particular region of the brain on the transmission and exchange of information within extensive networks of the brain) in SN regions, such as the anterior insula (AIns) and dorsal anterior cingulate cortex (ACC) ^13–15^.

Despite extensive research conducted on the topic, there is still insufficient evidence of any effects of ELS-related events on alterations of brain connectivity of salience, executive, somatosensory and impulse control networks in individuals with AUD, especially with varying BMI levels. In our earlier study using data from the human connectome project (HCP) we found notable associations between chronic alcohol consumption, BMI and the decision-making capabilities for monetary rewards in people with high-risk AUD and obesity symptoms ^16^. Expanding on these findings, our current objective is to examine how BMI modulates the impact of ELS on resting state seed-based functional connectivity among individuals with AUD. Our hypothesis is that ELS may influence the reduced connections of the salience network node with regions responsible for executive control and decision making in AUD, with patients with overweight or obesity (BMI ≥ 25 Kg/m^2^) and with AUD showing more pronounced effects then individuals with leanness (BMI ≤ 24.9 Kg/m^2^) and AUD.

## Materials and methods

### Participants

The study cohort, as presented in Table 1, was comprised of 401 treatment-seeking individuals with AUD (33% females) of which 123 individuals underwent resting state functional MRI scans. These were inpatients admitted in the NIAAA clinic for treatment and their resting state functional MRI scans were obtained following detoxification and withdrawal period from alcohol. Diagnosis of AUD was made via the Structured Clinical Interview for the Diagnostic Statistical Manual (DSM)-IV or DSM-5 (SCID)^17–19^. For the current analysis, individuals who were diagnosed with alcohol dependence or abuse via SCID-IV were considered to have an AUD. Daily alcohol consumption in the 90 days preceding the study was assessed using the timeline follow-back (TLFB) method ^20^. Individuals with substance use disorders other than AUD and nicotine dependence were excluded. Patients were allowed to smoke during their stay but were requested not to smoke or remove their nicotine patch two hours prior to their MRI scan. Of the individuals with AUD included in the present analysis, only 1% and 5% were found to experience alcohol-induced anxiety and mood disorders, respectively. The study was approved by the Institutional Review Board of the National Institutes of Health, and all participants provided written informed consent to participate.

### Body-mass index (BMI)

BMI for all participants in the study was calculated by dividing their body weight by the square of their height (Kg/m^2^). Measurements of both body weight and height were recorded when participants enrolled in the NIAAA natural history study. The continuous BMI variable was utilized to categorize the AUD cohort into two groups: individuals with a low BMI (BMI ≤ 24.9) and those with a high BMI (BMI ≥ 25). Categorizing the AUD cohort into low BMI and high BMI groups allows for a more nuanced analysis of the relationship between BMI-, AUD- and ELS-influenced brain connectivity patterns.

### Early life stress (ELS) events

ELS was operationalized using self-report questionnaire which consists of 19 standard life event items experienced in their early life as a child (up to age 18 years). The participants’ responses were recorded as yes/no for each life event including emotional, sexual and physical abuse, as well as violence, negligence, parental divorce, surgery, parental death, separation and so forth ^21, 22^. The sum of responses for all domains was used to create a complete ELS_events score (with a maximum score of 19; *Mean* = 3.58, *SD* = 3.2). Emotional abuse, severe family conflict, domestic violence and bullying were the most reported events.

### Resting-state functional MRI (rsfMRI) data acquisition and preprocessing

Resting-state fMRI (rs-fMRI) scans were acquired from patients during the inpatient treatment phase when they were stabilized and not experiencing stress or acute withdrawal symptoms. Patients’ withdrawal scores were assessed using the Clinical Institute Withdrawal Assessment for Alcohol-revised (CIWA-Ar), a 10-item scale ^23^. To be eligible for rs-fMRI scans, patients had to have a average CIWA-Ar score below 8, which typically occurs at 1 week after admission, and the scans were done during weeks 2 or 3 of their inpatient stay. The scans were conducted at the NIH NMR Center, utilizing a Siemens 3T MRI Skyra scanner with a 20-channel head coil. Participants were instructed to keep their eyes open and remain alert during the ten-minute period of rs-fMRI data collection, with no additional stimuli presented. The functional scans, including the rs-fMRI, were acquired utilizing an echoplanar-imaging pulse sequence (TR: 2000 ms, TE: 30 ms, FA: 90°, FOV: 240 × 240 mm, 3.8 mm slice thickness, multi-slice mode: interleaved). A high-resolution T1-weighted MPRAGE (TR: 1900 ms, TE: 3.09 ms, FA: 10°, FOV: 240 × 240 mm, 1 mm slice thickness) was obtained for registration purposes. Preprocessing of the data was carried out using the CONN toolbox (version 18b), a Matlab-based toolbox for functional connectivity analysis (http://www.nitrc.org/projects/conn) ^24^, including realignment and unwarp, slice-timing correction, outlier identification, and normalization. Artifact detection was performed based on scan-to-scan differences in the global signal (z-value threshold 5) and subject motion parameters (threshold 0.9 mm) using the ‘art’ software for artifact rejection (www.nitrc.org/projects/artifact_detect/), with identified outlier scans included as first-level covariates.

### Functional connectivity

To analyze rs-fMRI data, we used the CONN toolbox (18.b; https://www.nitrc.org/executedashboard/?group_id=279) with full width at half maximum spatial smoothing of 8 mm. To minimize effects of head motion, we regressed out principal components associated with segmented white matter and cerebrospinal fluid using CompCor ^25^, as well as twelve motion regressors (3 rotational, 3 translational, and their derivatives) calculated from CONN image preprocessing. The data were filtered using a band-pass filter of 0.008–0.09 Hz to eliminate very-low-frequency drift and high frequency noise, and linear trends were removed. We used a continuous squashing function (*i.e.*, despiking) to further minimize the influence of potential outlier scans. Global BOLD signal was not regressed out to avoid the mathematical introduction of negative correlations ^26^.

We conducted a seed to voxel (whole brain) resting state connectivity analysis to investigate the influence of ELS on the connectivity of SN seed regions and the rest of the brain. The seeds were selected *a priori* based on our hypothesis of strong interaction of SN with executive function networks in addictive conditions ^27–29^. The seeds were defined based on the anatomical FSL Harvard-Oxford atlas, which is the default atlas utilized for segmentation during the CONN processing procedure. We included the anterior insula, anterior cingulate cortex and inferior parietal cortex supramarginal gyrus as seed regions associated with the SN. A separate model was created for the left and right structures for each seed. We extracted the mean time series of the seed region from their preprocessed functional data and calculated Pearson’s correlation coefficients for the connection between the seed and voxel for each participant. To enable further analyses, we transformed the resulting values into normally distributed Z-scores using the Fisher transformation. The identified correlations are presented in the results section.

### Statistical analyses

**Figure 1:**
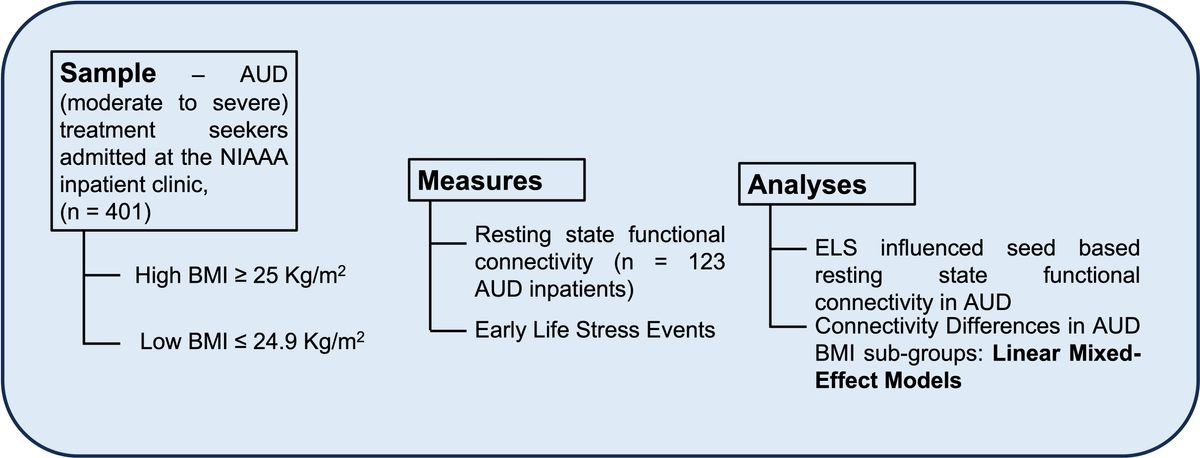
The study flow listing the sample, measures and analytic techniques. The alcohol use disorder (AUD) population was divided into two groups based on body-mass index (BMI) (High BMI and Low BMI). The measures included early life stress (ELS) events and resting-state functional connectivity (rs-fMRI). The main analyses consisted of seed-based rs-fMRI to identify early life stress-influenced connectivity patterns in individuals with AUD, as well as linear mixed-effects models to identify connectivity differences in the AUD BMI groups.

To compare the demographic and clinical characteristics of our study groups (AUD: low BMI vs. high BMI), we utilized Student’s t-tests and Mann-Whitney tests for continuous variables, and chi-squared tests for categorical variables. To identify group differences, ELS-associated connectivity coefficients between the seeds and significant clusters were extracted from CONN. We considered connection-level False Discovery Rate (FDR)-corrected P-values < 0.05 as significant ^30^. We then employed linear mixed effect models (LMEs) to investigate differences in correlations between the seed and significant clusters (interpreted as connectivity, the dependent variable) across our study groups and included AUDIT, BMI status, age, sex, and smoking status as fixed effects, and participants as random effect. We adjusted for multiple pairwise group comparisons using the Bonferroni correction. For all descriptives and regression models, we used SPSS 22 (IBM Corp., Armonk, NY) and addressed missing values in LMEs using the restricted maximum likelihood approach. Figure 1 provides an overview of the study flow, including participant grouping, measures and statistical approach used to investigate the study hypothesis.

## Results

### Characteristics of the sample: demographic and clinical

Of a total of 401 participants with AUD (age mean SD: 46.21 ± 11.48; range: 21–74 yrs), *n* = 240 (60%) had a BMI ≥ 25 kg/m^2^ (AUD-high BMI), while *n* = 161 (40%) had a BMI between 18–24.9 Kg/m^2^ (AUD-low BMI). The resting state fMRI scans were acquired in 123 of the total participants [(age mean SD: 28.16 ± 6.28; range: 25–69 yrs), *n* = 71 AUD-high BMI; *n* = 52 AUD-low BMI]. The proportion of males was higher in the high BMI group (76.1%) compared to the low BMI group (56.6%; χ2 = 5.25; p = 0.02). The length of AUD history (difference in age of AUD onset and current age) was significantly higher among participants with a high BMI *vs*. those with a low BMI (p = 0.02). The full BMI distribution of the AUD cohort and sub-cohort (patients that underwent rs-fMRI) can be seen in **Supplemental Figures S1 and S2**. The data show a remarkable increase in rightward tail for BMI distribution in both the samples. However, there was no clear divergence in the occurrence of their ELS events between our BMI groups.

In the AUD cohort, the mean age of high BMI group (47.9 ± 11.3 years) was significantly higher than the low BMI counterpart (43.7 ± 11.3 years; *p* < 0.001). Furthermore, the percentage of smokers was higher in the low BMI (77.6%) category then the high BMI (54.6%; *p* < 0.001). No significant difference in sex, race, ethnicity, years of education and household income was identified between the BMI groups (refer to Table 1).

The BMI groups in the AUD sub-cohort exhibited no differences in age, sex, race, ethnicity, years of education, household income or smoking status. As expected, the two AUD BMI groups did not differ in their age of first alcohol drink (refer to Table 1).

## Resting state fMRI

### Influence of ELS events on seed-based voxel connectivity (SBVC)

**Figure 2:**
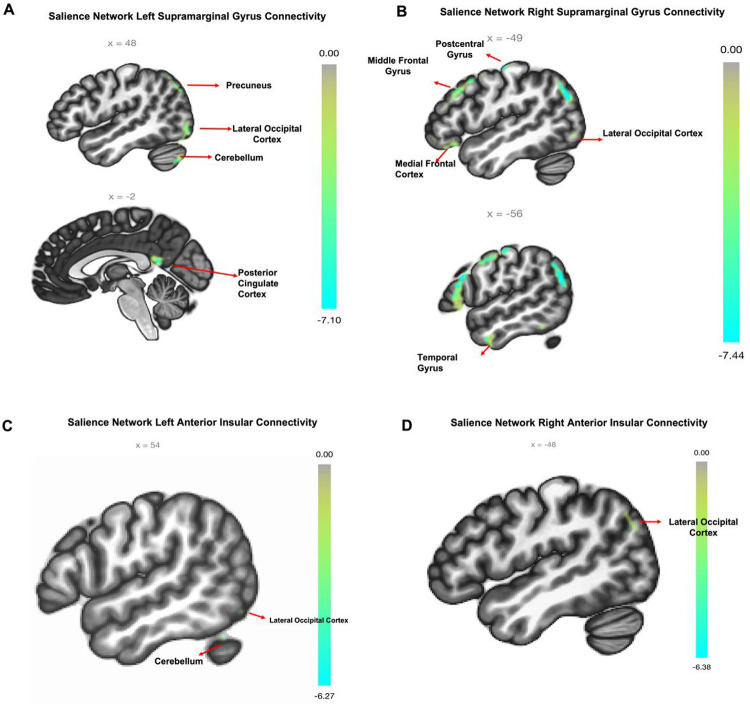
Early life stress-related connectivity pattern in AUD. (A – D) Significant Seed-to-Voxel Connections Representing Salience Network Seed Regions (supramarginal gyrus left/right and anterior insula left/right). For anterior cingulate connectivity: plots of seed connectivity are not pictured as cluster size was small. Labels provided for perspective reference are as follows: LOC: Lateral Occipital Cortex, PCC: Posterior Cingulate Cortex, mFC: medial frontal cortex, PoG: Postcentral Gyrus, Ins: Insula, MFG: Middle Frontal Gyrus, MTG: Middle Temporal Gyrus; cluster of significant activation at the peak-wise PFWE < 0.001/cluster size P < 0.05 FDR corrected level. Directions of connectivity are noted in Table 2.

The occurrence of ELS events predicted connectivity of multiple clusters to our seed regions; namely, left/right SMG, AIns and ACC after covarying for age and sex. For further information on the connected clusters, please refer to Table 2. Additionally, a visual representation of the extent of significance can be found in Fig. 2.

Left SMG showed negative connectivity driven by ELS with several clusters, including the right lateral occipital cortex (LOC), right cerebellum and left intracalcarine cortex, as well as the posterior cingulate cortex (PCC). However, left SMG had positive connectivity with clusters in the precuneus and left frontal pole. Conversely, driven by ELS the right SMG showed a positive connectivity with the left insular cortex and right cerebellum 8 clusters, but it was negatively connected to clusters in the frontal medial cortex, left postcentral gyrus, left middle frontal gyrus (MFG), left LOC, left middle temporal gyrus (MTG) and right cerebellum crus 2.

The ACC seed region exhibited positive connectivity with a cluster in the left anterior temporal fusiform cortex (ATFC). As for the left AIns seed, it showed negative connectivity with clusters in the right LOC and Cerebellum Crus 1. Meanwhile, the right AIns seed was negatively connected to the left LOC but positively connected to the right hippocampus.

### Association of ELS predicts seed-based voxel connectivity with BMI levels in individuals with AUD

**Figure 3:**
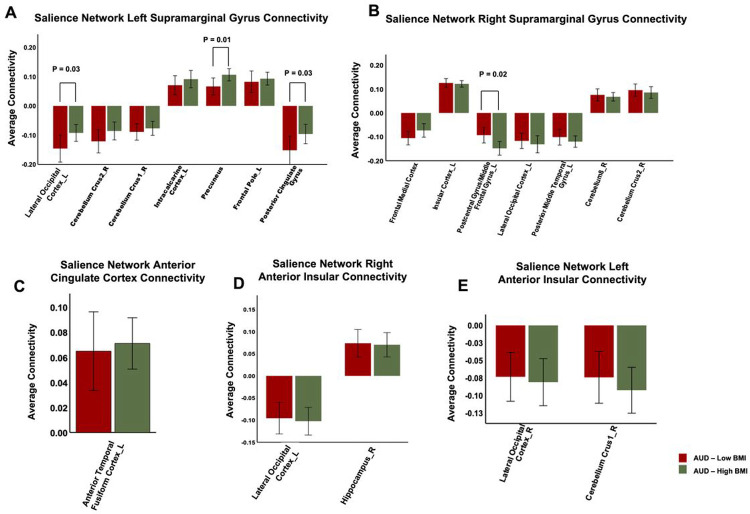
ELS-Driven Differential Seed-to-Voxel Connectivity Patterns: BMI Effects on AUD Groups. The bar graphs illustrate the differential seed-to-voxel connectivity patterns driven by early life stress (ELS), focusing on the effects of BMI on AUD groups. Linear mixed effect models were utilized, adjusting for age, sex, and smoking status. The upper panel (A/B) displays the connectivity coefficients of the Supramarginal Gyrus (Left/Right) seeds for the respective groups. The lower left panel (C) represents the connectivity coefficient of the Anterior Cingulate Cortex seed by group. The lower middle panel (D) shows the connectivity coefficient of the Right Anterior Insula seed by group. Lastly, the lower right panel (E) exhibits the connectivity coefficient of the Left Anterior Insula seed by group. The provided labels serve as reference: AUD (Alcohol Use Disorder), BMI (Body-Mass Index), ELS (Early Life Stress), L (Left), R (Right). Multiple columns of bar graphs represent various significant clusters identified by name. Significance levels (p < 0.05) are indicated after Bonferroni correction.

Our LME analysis, after adjustment for age, sex and smoking status, showed that individuals with AUD and low BMI (in comparison to those with high BMI) exhibited several negative correlations, such as in connectivity of the left SMG seed with the right LOC (β = −0.06; CI = −0.12, −0.002; *p* = 0.03) and PCC (β = −0.05; CI = −0.114, −0.002; *p* = 0.03) clusters. In addition, they also displayed a negative correlation with the connectivity of the left SMG with the precuneus (β = −0.03; CI = −0.07, −0.005; *p* = 0.01) cluster (refer to **Table 3**; Fig. 3A).

***Further investigation showed that low BMI individuals revealed anticorrelation of right SMG seed connectivity with the left postcentral gyrus/MFG cluster (β = 0.04; CI = 0.005, 0.089; p = 0.02) compared to those with high BMI (Table 2; Fig. 3B). However, we did not observe any significant differences between BMI groups in the connectivity patterns of the ACC and AIns seeds with different brain clusters in the AUD BMI groups (refer to Table 2, Fig. 3C, D & E).

## Discussion

In our population of individuals with moderate-to-severe AUD, we found a higher prevalence of individuals with overweight and obesity (approximately 60%). However, when examining the distribution of BMI, we did not observe a significant rightward skewing, indicating a relatively balanced distribution across the range. Surprisingly, we did not find a clear difference in the distribution of ELS events between patients with AUD and with a high or low BMI. Additionally, as we identified a significantly greater length of AUD history in participants with a high BMI as compared to a low BMI within our cohort, implying that those with a low BMI are still early in their history of AUD and have yet to gain weight. These findings suggest that the relationship between BMI and ELS in the context of AUD may not be straightforward, and there may be other factors that have a stronger impact on the association between ELS and BMI in individuals with AUD.

Therefore, on investigating the effect of ELS on functional brain connectivity in individuals with AUD who differ in BMI, we observed various connectivity patterns associated with ELS, indicating connections between the left and right SMG seed regions and whole brain. Also, these predicted connections were differentially associated with BMI levels within these individuals.

As predicted, we observed the main effect of ELS as anticorrelation of the SN seed regions, bilateral SMG and AIns, with several clusters in different regions of the brain, including the somatosensory and motor coordination areas (such as the bilateral LOC, right cerebellum, left posterior middle temporal gyrus (pMTG), left postcentral gyrus, and left intracalcarine cortex), frontal, or executive, control regions (*e.g.*, the MFG). Exposure to stress during critical periods of brain development has been demonstrated to modify connectivity patterns and heighten the risk of developing AUD. Likewise, numerous studies provide evidence that experiencing ELS has harmful effects on individuals and enhances their susceptibility to alcohol use in adulthood ^2, 31–35^. Moreover, exposure to a series of ELS events leads to modifications in connectivity of brain regions associated with emotion, self-regulation and cognition, including nodes of the DMN, such as the PCC, mPFC and MTG, and within the fronto-limbic networks, such as the mPFC, ACC, amygdala and orbitofrontal cortex ^36–38^]. Children between the ages of 9 and 16 who were exposed to various stress events, such as conventional crimes, child maltreatment, peer/sibling victimization and sexual victimization, were found to have a reduced functional connection between their SMG and PCC ^39^. Studies have shown that in individuals with AUD, reduced connectivity within the SN is associated with decreased self-control and an inability to restrain cravings ^5^, as well as impaired inhibition for salient stimuli ^40^. Notably our analysis revealed lower functional connectivity between left/right SMG and clusters in motor [cerebellum (right Crus 1 & 2), occipital cortex, temporal (pMTG)] and somatosensory (postcentral gyrus), as well as frontal, or executive, control regions (*e.g.*, the MFG) that reportedly are involved in processing of emotionally salient stimuli ^41–44^. As such, the lower connectivity of SN nodes with emotion and executive control regions are concordant with evidence in the literature that have shown the effect of ELS exposure in altering the connectivity of brain networks involved in emotion regulation and salience of rewards ^45, 46^. These previous findings suggest a disengagement of executive control functions when emotionally significant rewards are being processed in adults with AUD and a history of ELS. The negative association between left SMG activity and major default mode network (DMN) nodes (PCC, mPFC), which are normally inversely associated with goal-oriented actions ^47^, suggests that ELS can affect the decision-making ability of individuals with AUD. Such altered emotional processing and decision-making, resulting from ELS, may be interpreted as the underlying drivers for the development of stress-related AUD ^48^.

Moreover, we found that the functional connectivity of SN seeds was influenced by ELS, resulting in a positive correlation between the left SMG and ACC with DMN nodes (precuneus and left ATFC) in the AUD population. It is noteworthy that exposure to acute stress has been linked with elevated functional connectivity between the default mode and salience networks in healthy adults and adolescents ^39, 49^. Given that the participants in our study cohort were exposed to a variety of ELS events, spanning from one to nineteen stress events, it is probable that the direction of connectivity between different regions of the network was impacted by the overall degree of ELS they experienced. Our findings align with previous research suggesting that certain nodes within the DMN, such as the temporal fusiform cortex and precuneus, play a crucial role in social and self-related cognitive processes ^50, 51^, supporting the interpretation that increased SMG-ATFC/precuneus coupling, as a result of ELS events, may lead to heightened self-awareness and emotional response to negative social stimuli. This, in turn, could potentially increase impulsive decision-making and drinking behaviors as a way of regulating these emotions in individuals with AUD.

We further investigated the impact of BMI on ELS-influenced connectivity patterns of the SN with different brain regions in AUD. Contrary to our expectations, individuals with AUD and low BMI exhibited a stronger anticorrelation in the connectivity of the left SMG seed with the right LOC and PCC clusters, while exhibiting a weaker positive correlation with the precuneus cluster, compared to those with high BMI. This suggests that the SN left SMG connectivity with emotion regulation and decision-making regions is not always negatively impacted by AUD and may even be strengthened in these individuals with a low BMI. On the other hand, we observed that the ELS influenced anticorrelation of the right SMG seed with the somatosensory (left postcentral gyrus) and impulse control (left MFG) clusters was weaker in AUD-low BMI individuals compared to those with high BMI, which in light of previous findings ^27, 28, 52–54^, suggest elevated impulsivity and poor self-control behaviors in AUD with co-occurrence of high BMI. The findings suggest two potential scenarios: either ELS contributes to overeating in individuals with AUD, or ELS-induced overeating increases their susceptibility to excessive alcohol consumption.

Upon further examination, we found that the influence of ELS on connectivity patterns with the left and right hemispheric SN SMG seeds differed based on individuals’ BMI levels within our population with AUD. In particular, the magnitude of increase in BMI levels were observed to exert an impact on the connectivity of the left SMG with regions associated with the default mode network. On the other hand, a negative correlation was detected between the BMI levels of individuals with AUD and the connectivity of the right SMG with clusters in frontal regions that govern impulsive or self-control behaviors. Both pre-clinical and clinical studies have identified the contribution of ELS in increasing the risk for obesity ^55–57^ and AUD ^32, 58, 59^, which was attributed to persistent overactivation of the hypothalamic-pituitary-adrenal (HPA) axis ^60^, dysregulation of the mesolimbic dopamine functions ^61, 62^ and an imbalance in connectivity patterns of salience, emotion and somatosensory networks ^63^. Nonetheless, none of these studies demonstrated the relationship between ELS-influenced brain connectivity in adults with a comorbid occurrence of AUD and elevated BMI.

The present study offers intriguing insights into the intricate relationship between ELS, AUD, BMI and the connections of salience network seeds with the entire brain. These results are consistent with our hypothesis, which suggests that elevated BMI may alter the connectivity between the SN and brain regions that regulate executive and impulsive behaviors in individuals with AUD who have a history of ELS.

## Limitations

There are several unanswered questions that need to be explored in future large cohort studies. One limitation of our study is the use of self-reported questionnaire to measure ELS events. This type of measure is prone to recall bias and may not provide a complete evaluation of ELS. Furthermore, our study did not disentangle the effect of each type of ELS experience, even though research shows that different adverse events may have different effects on brain structure and network connectivity. For example, deprivation and neglect are linked to changes in executive control network regions, such as the dorsolateral prefrontal cortex and parietal cortex, while threat and abuse-related exposures are linked to alterations in regions of the salience network ^64^. Additionally, adults who grew up in poverty exhibit reduced activation in the ventrolateral prefrontal cortex and have difficulty regulating emotions ^65^. In a recent study alterations in connectivity within the SN was found to mediate the effects of childhood abuse and neglect with problematic alcohol use ^66^. Although there are no studies that have directly compared the impact of ELS on connectivity differences based on BMI levels in the AUD population, the age at which the stress occurred ^67^ and the level or duration of stress exposure ^68^ are crucial factors that should be explored in future studies. Moreover, the correlations observed between ELS and brain connectivity in individuals with AUD at different BMI levels give rise to various conclusions. For instance, it is possible that ELS influences both alcohol abuse and excessive eating. Alternatively, it could be that ELS-driven AUD contributes to overeating, or that ELS-driven overeating increases vulnerability to alcohol overconsumption. The significance of these findings emphasizes the need for longitudinal studies instead of solely relying on cross-sectional research. It is also crucial to conduct longitudinal studies that follow individuals with AUD from an early stage, allowing the observation of potential changes in their brain patterns over time, particularly in relation to any fluctuations in BMI. Lastly, our study did not identify connectivity differences with salience network seeds in AIns and ACC, as reported in previous studies on the effects of ELS on salience network seed-based connectivity. This may be attributed to the relatively low severity of the stressors reported in our cohort, making direct comparisons with previous results difficult. In our forthcoming study, we aim to investigate potential sex effects that may be influencing the observed correlations and relationships. Furthermore, it is crucial to replicate our findings using large datasets to assess the consistency and reliability of the results, reinforcing the significance and validity of our study’s outcomes.

## Conclusion

To conclude, we identified positive and negative correlations in the connectivity of our SN SMG seed with clusters associated with emotion, self-regulation, decision-making and impulsivity for salient stimuli in an AUD population with a history of ELS-related events. Further our results revealed the impact of BMI elevation on the observed brain connectivity patterns in study participants with AUD. These findings underscore the significance of ELS-induced SN seed connectivity in AUD with varying BMI levels and its role in the neurobiological mechanisms that drive AUD. The results from our study suggest potential directions for future large-scale research on the neural mechanisms for the comorbid occurrence of obesity in individuals with AUD with a history of ELS. This might facilitate the development of targeted interventions for such individuals.

## Figures and Tables

**Table 1:** Demographic and Clinical Characteristics of Participants

	AUD cohort (n = 401)			AUD sub-cohort (n = 123)		
	High BMI (n = 240)	Low BMI (n = 161)	Test Statistics T-test or, Mann-Whitney for continuous/Chi-square for categorical variables	High BMI (n = 71)	Low BMI (n = 52)	Test Statistics T-test or, Mann-Whitney for continuous/Chi-square for categorical variables
*Age (years) (mean ± SD)*	47.9 ± 11.3	43.7 ± 11.3	p < 0.001	45.4 ± 11.2	43.5 ± 10.6	p = 0.33
*Sex*						
Males n (%)	163 (67.9%)	106 (65.8%)	χ^2^ = 0.19; df = 1; p = 0.66	54 (76.1%)	30 (56.6%)	χ^2^ = 5.25; df = 1; p = 0.02
Females n (%)	77 (32.1%)	55 (34.2%)		17 (23.9%)	23 (43.4%)	
*Years of Education*	13.7 ± 2.8	13.6 ± 3.1	p = 0.78	13.4 ± 2.9	13.4 ± 2.5	p = 0.94
*Household Income (Median)*	30K-39 999	30K-39 999		30K-39 999	30K-39 999	
*Total drinks last 7 days*	92.42 ± 67.02	100.63 ± 88.27	p = 0.29	91.83 ± 64.87	87.28 ± 68.50	p = 0.74
*Alcohol Dependence Score*	21.22 ± 8.36	21.93 ± 8.58	p = 0.41	21.52 ± 7.98	22.73 ± 8.43	p = 0.41
*Age First Drink [years (mean ± SD)]*	14.76 ± 4.00	14.79 ± 5.24	p = 0.94	14.64 ± 3.64	14.56 ± 4.64	p = 0.91
*Age of AUD Onset [years (mean ± SD)]*	28.7 ± 10.59	27.3 ± 10.03	p = 0.21	25.95 ± 9.10	27.64 ± 9.08	p = 0.33
*Length of AUD history*	19.26 ± 11.95	16.38 ± 10.95	p = 0.02	19.08 ± 11.84	15.79 ± 9.23	p = 0.19
*(mean ± SD)*						
*Smoking status*						
Yes, n (%)	131 (54.6%)	125 (77.6%)	χ^2^ = 22.19; df = 1;p < 0.001	42 (59.2%)	36 (67.9%)	χ^2^ = 1.0; df = 1; p = 0.32
No, n (%)	109 (45.4%)	36 (22.4%)		29 (40.8%)	17 (32.1%)	
*Ethnicity, n (%)*						
Non-Hispanic or Latino	214 (89.2%)	143 (88.8%)	χ^2^ = 2.26; df = 2; p = 0.32	61 (85.9%)	48 (90.6%)	χ^2^ = 0.62; df = 2; p = 0.73
Hispanic or Latino	20 (8.3%)	10 (6.2%)		8 (11.3%)	4 (7.5%)	
Unknown/not reported	6 (2.5%)	8 (4.9%)		2 (2.8%)	1 (1.9%)	
*Race, n (%)*						
White	127 (52.9%)	78 (48.4%)	χ^2^ = 5.31; df = 5; p = 0.38	37 (52.1%)	30 (56.6%)	χ^2^ = 2.89; df = 5; p = 0.71
Black/African American	83 (34.6%)	63 (39.1%)		21 (29.6%)	15 (28.3%)	
Asian	3 (1.3%)	5 (3.1%)		1 (1.4%)	0	
American Indian or Alaska Native	4 (1.7%)	0		1 (1.4%)	0	
Multiracial	7 (2.9%)	5 (3.1%)		4 (5.6%)	5 (9.4%)	
Unknown Race	16 (6.7%)	10 (6.2%)		7 (9.9%)	3 (5.7%)	
ELS events (Median; IQR)	3; 5	3; 5		2; 4	3; 5	

Note: AUD cohort: NIAAA AUD inpatient treatment seekers; AUD sub-cohort: AUD inpatients who underwent resting state functional MRI;

NA: not available; Household Income: <$10,000 = 1, $10K–$19,999 = 2, $20K–$29,999 = 3, $30K–$39,999 = 4, $40K–$49,999 = 5, $50K–$74,999 = 6, $75K–$99,999 = 7, >=$100 000 = 8; length of AUD history (difference in age of AUD onset and current age). Here, * represents statistical significance with p value < 0.05; only white and black populations were included for chi-square analysis for racial differences.

**Table 2:** ELS_events Effect and Severe AUD - BMI Group Differences in the Seed-to-Voxel Analysis

Seed		Connectivity Cluster				Adjusted Estimate (β), (95% CI), p value			
Label	Lat.	Label	Lat.	x	y	z	k	P (peak, FWE)	Connectivity differences between AUD – BMI groups (AUD Low BMI vs. AUD High BMI)
Salience Network_SMG	L	Lateral Occipital Cortex	R	+52	−66	+40	2584	<0.001	−0.06, (−0.12, −0.002), 0.03*
		Cerebellum Crus 2	R	+46	−68	−44			−0.037, (−0.08, 0.015), 0.17
		Cerebellum Crus 1	R	+48	−74	−20			−0.016, (−0.05, 0.019), 0.36
		Intracalcarine cortex	L	−08	−76	+12			−0.022, (−0.06, 0.02), 0.31
		Precuneus		+14	−74	+40	145	0.01	−0.039, (−0.07, −0.005), 0.01*
		Frontal Pole	L	−26	+40	−16	89	<0.001	−0.01, (−0.05, 0.029), 0.59
		Posterior Cingulate Gyrus		−02	−52	+18	61	0.003	−0.05, (−0.114, −0.002), 0.03*
	R	Frontal Medial Cortex		−02	+32	−24	726	0.02	−0.033, (−0.07, 0.007), 0.105
		Insular Cortex	L	−52	−32	+22	8616	<0.001	0.002, (−0.02, 0.02), 0.18
		Postcentral Gyrus	L	−56	+20	+26	1011	<0.001	0.04, (0.005, 0.089), 0.02*
		Middle Frontal Gyrus	L						
		Lateral Occipital Cortex	L	−52	−70	+32	539	<0.001	0.01, (−0.03, 0.06), 0.62
		Posterior Middle Temporal Gyrus	L	−66	−44	+08	362	<0.001	0.015, (−0.02, 0.05), 0.43
		Cerebellum8	R	+36	−48	−54	206	<0.01	0.004, (−0.02, 0.03), 0.77
		Cerebellum Crus2	R	+52	−50	−42	45	<0.001	0.008, (−0.02, 0.04), 0.65
Salience Network_ACC		Anterior Temporal Fusiform Cortex	L	−36	−04	−42	143	0.004	−0.008, (−0.04, 0.02), 0.63
Salience Network_AIns	L	Lateral Occipital Cortex	R	+46	−78	−20	119	<0.01	0.011, (−0.03, 0.06), 0.64
		Cerebellum Crusl	R	+54	−58	−28	74	0.03	0.016, (−0.03, 0.06), 0.51
	R	Lateral Occipital Cortex	L	−50	−70	+32	230	<0.001	0.007, (−0.039, 0.054), 0.75
		Hippocampus	R	+32	−22	−20	41	0.023	−0.001, (−0.042, 0.039), 0.94

**Notes:** Statistics and descriptives for seed-to-voxel connectivity analyses. Abbreviations as follows: k: number of voxels in the cluster; Lat. (R/L): Laterality of brain region (Right/Left); FDR: false discovery rate correction; SD: standard deviation; SMG = Supramarginal Gyrus; AG = Angular Gyrus; ACC = Anterior Cingulate Cortex; AIns = Anterior Insula; AUD: Alcohol Use Disorder; BMI: Body-Mass Index. Group differences were adjusted for age, smoking status.

## Data Availability

The information analyzed in this study from the NIAAA is bound by specific licenses and restrictions. The dataset is under the care and control of the NIAAA Office of the Clinical Director and is securely housed there. Dataset access requests are directed to Melanie Schwandt, melanies@mail.nih.gov.
